# Parylene C topographic micropattern as a template for patterning PDMS and Polyacrylamide hydrogel

**DOI:** 10.1038/s41598-017-05434-6

**Published:** 2017-07-18

**Authors:** Ilaria Sanzari, Mauro Callisti, Antonio De Grazia, Daniel J. Evans, Tomas Polcar, Themistoklis Prodromakis

**Affiliations:** 1 Nanoelectronics & Nanotechnology Research Group, Department of Electronics and Computer Science, Faculty of Physical Science and Engineering, University of Southampton, University Road, Southampton, SO17 1BJ United Kingdom; 2 Engineering Science, University of Southampton, Highfield Campus, Southampton, SO17 1BJ United Kingdom; 3 Department of Electronics and Computer Science, University of Southampton, Highfield Campus, Southampton, SO17 1BJ United Kingdom

## Abstract

Parylene C is a well-known polymer and it has been mainly employed as a protective layer for implantable electronics. In this paper, we propose a new approach to use Parylene C as a versatile template for patterning soft materials potentially applicable as scaffolds in cardiac tissue engineering (TE). Parylene C substrates were anisotropically patterned through standard lithographic process with hydrophilic channels separating raised hydrophobic strips. Ridges and grooves of the template are 10 µm width and depth ranging from 1 to 17 µm. Polydimethylsiloxane (PDMS) and Polyacrylamide (PAm) hydrogel have been chosen as soft polymers to be moulded. Thanks to their chemical and physical properties PDMS and PAm hydrogel mimic the extracellular matrix (ECM). PDMS was spin coated on micropatterned Parylene C obtaining composite substrates with 460 nm and 1.15 µm high grooves. The Young’s modulus of the composite Parylene C/PDMS was evaluated and it was found to be almost half when compared to PDMS. PAm hydrogel was also printed using collagen coated micro-grooved Parylene C. Optical micrographs and fluorescence analysis show the successful topographic and protein pattern transfer on the hydrogel.

## Introduction

Strategies for designing and fabricating synthetic polymers are having substantial interest especially in TE where the development of scaffolds is crucial for cell differentiation and growth^1, 2^. Scaffolds based on synthetic polymers serve as artificial supportive extracellular matrices mimicking native conditions where cell normally live. Specific cells are affected by different types of stimuli from ECM so the choice of one type of polymer rather than another is crucial in synthetic biology^3^. For instance, cardiomyocytes *in vivo* are surrounded by an elastic matrix with a Young’s modulus ranging from 10 kPa to maximum 1 MPa^4^. In addition, the anisotropic nanoscale topography of cardiac ECM promotes cell alignment and function^5^. Topography and elasticity effect on cardiomyocytes have been tackled using different polymers such as polyurethane acrylate^5^, Parylene C^6^, PDMS^7^ and PAm hydrogel^8^.

Parylene C is a chloro-poly-para-xylene polymer deposited via chemical vapor deposition (CVD). Thanks to its biocompatibility, chemical inertness and insulation performance it is widely employed in biomedicine especially as a protective encapsulation for neuronal prosthesis^9^ and cardiac implants^10^. In addition, Parylene C unlike PDMS is impervious to liquids and displays low permeability to gases, for this reason it was exploited in microfluidics devices as protective layer for PDMS to prevent liquid absorption^11^, bubble formation and liquid evaporation in micro-channels^12^. Application of Parylene C for cell culturing requires surface modification such as UV irradiation^13^, oxygen (O_2_) plasma^14^ or argon (Ar) plasma^15^. These treatments determine the oxidation of methylene groups forming carboxylic groups necessary for the hydrophilic interactions and adhesion bonds between biomolecules and polymer surface^16^. O_2_ plasma has been mainly studied demonstrating a very effective treatment in terms of hydrophilicity retention (Parylene C restores 40–50% of its hydrophobicity after a week^14^), cell adhesion and spreading^17^. Parylene C can also be etched from oxygen plasma^18^ becoming compatible with standard lithographic processes.

Different strategies have been applied to pattern Parylene C surfaces such as hydrophobic/hydrophilic grooved surface for mimicking cardiomyocyte ECM resulting in a significant change in calcium activity of cardiomyocytes at 0.5–2 Hz stimulation, mature phenotype and well aligned sarcomeres^6^. Thin films of Parylene C (2–10 µm thick) with hydrophobic/hydrophilic grooves 0.5 µm deep were also compared in order to understand cardiomyocyte morphology and activity. In this study, 10 µm thick substrates revealed higher microtubule density in cells than thin substrates 2 µm thick. Although Parylene C is mechanically robust with a high Young’s modulus (~3.2 GPa^19^), it does not mimic totally the stiffness of the most extracellular environment especially of muscular cells. For example, it has been demonstrated that cardiomyocytes on substrates stiffer than 10 kPa showed anomalous stress fibers not normally present *in vivo* ^20^. Although its attractive properties, Parylene C does not exhibit tunable stiffness like other polymers (e.g. PDMS, polyacrylamide, etc.) that can change the elastic properties modifying the ratio between the monomer and the cross-linker, consequently the use of this material has been mainly confined to packaging purposes.

In the last ten years, the mechanical robustness of Parylene C has been exploited using this material as a reusable mask to pattern either elastomers^21^ or proteins or cells on different substrates. For example bovine serum albumin and NIH-3T3 fibroblasts were patterned on glass, polystyrene and PDMS using pinhole Parylene C masks^22^. Thicker Parylene C microstencils (10–20 µm) were fabricated through standard lithographic processes and Parylene C masks were bonded in the surface, coated with biomolecules or cells, and later peeled off from the surface. With this technique wide areas (~50 µm of diameter) were patterned and high material thickness was required in order to avoid the low tensile strength of thin Parylene C films that usually tore apart during the lift off. PDMS was also topographically patterned via Parylene C masks to create microlens arrays^21^. PDMS was spin coated on Parylene C masks with holes of 50 µm of diameter obtained by etching. PDMS was spin coated on Parylene C and after curing it was manually peeled off leaving the PDMS only in the areas where the mask was opened. The dimensions and size of PDMS structures were checked at different curing time of PDMS, using different thickness of Parylene C films and specific heights of trenches hollowed in Parylene C mask. Although this method can provide thin PDMS layers and controllable shapes it presents two main shortcomings. First, hand-peeling of the mask is not a reproducible method since there is a continuous risk to break the mask. Secondly it is limited to certain types of design since the bilayer Parylene C/PDMS has to be removed together at the same time during the peeling off, the PDMS needs to cover connected areas^23^. Another common way for PDMS patterning is soft-lithography^7^ whose main drawbacks are related to the specific treatment of the molds in order to easily peel off the replica and to the not accurate control of the thickness of the PDMS. In addition, thin PDMS films are very tricky to peel off and Parylene C as a supportive substrate could help for this purpose and have a new soft hybrid membrane with anisotropic pattern on the surface.

Patterning of hydrogels is more straightforward than elastomers but more important for cell culturing applications. Thanks to their chemical and mechanical properties hydrogels mimic the ECM *in vitro* and can be excellent materials for scaffolds in TE. They are mostly obtained by radical polymerization or UV cross-linking. In dry condition they start to shrink losing water by evaporation. Therefore the main difficulty is to adapt hydrogels to classical microfabrication processe. Topographical patterning of PAm hydrogels has been proposed using micro contact printing based on PDMS^﻿24, 25^ or optical lithography with gel in dry condition or using UV cross-linking^26^. Even if these techniques allow good resolution (up to 2 µm^25^) they results in a non-uniform patterning mainly in the case of UV cross-linking. Moreover ECM proteins (e.g. collagen, laminin, fibronectin) have been covalently bounded directly on PAm hydrogel using different strategies such as cross-linking the surface by the heterobifunctional N-sulphosuccinimidyl-6-(40-azido-20-nitrophenylamino) hexanoate (Sulfo-SANPAH)^27^, covalent modification of non-reactive amide groups to reactive hydrazide groups using hydrazine hydrate^28^ and chemical modification of the composition of the precursor solution of the hydrogel through the incorporation of hydroxyl groups^29^. These procedures are very time consuming, required toxic chemicals and refined chemistry strategies that could modify the physical properties of the hydrogel^29^.

In this paper we demonstrate how to pattern PDMS and PAm hydrogel topographically via Parylene C as a bottom mask for PDMS and top mask for PAm hydrogel. PDMS wrinkled and anisotropic surface at nano and microscale was obtained using simple spin coating and avoiding complicated techniques actually proposed such as soft lithography^7^ or elastomer pre-stretching^30^. Parylene C masks fabricated as described previously^6^ were exploited for a new technique to pattern PAm hydrogel. We have demonstrated that Parylene C selectively modified and coated with protein can be used as mould for patterning PAm hydrogel both topographically and biochemically using the robustness, the chemical resistivity and the selective pattern of Parylene C and making the process low cost and safe.

## Materials and Methods

### Fabrication of Parylene C masks and Parylene C/PDMS constructs

Parylene C templates for PDMS coating were fabricated with high aspect ratio micro-grooves (height of features of 17 µm). 25 µm thick Parylene C was deposited on 6 inch silicon wafers (Fig. 1a) followed up by a sputter coating of 200 nm thick aluminium (Al) (Fig. 1b). Parylene C was deposited via CVD using a commercially available Parylene coater (Labcoater PSD2010, SCS). The deposition of Parylene consists of three main steps. Firstly para-xylene dimer is vaporized at a temperature of 150 °C. Secondly the dimer is vaporized and the gas moves into the pyrolysis chamber where the dimer is converted into monomer at 690 °C. Finally the monomer moves into the deposition chamber where it is deposited on the surfaces. Optical lithography was used to pattern the surface (Fig. 1c). 1.3 µm thick positive photoresist (S1813, Microchemicals) was spin coated on Parylene C. Wafers were then soft baked at 110 °C for 60 s and exposed to UV light (EVG 620 Mask Aligner) for 2 s through a chrome-plated glass mask consisting of 1 cm × 1 cm repeated squares containing transparent lines equally spaced 10 µm wide and 10 µm apart. The samples were developed in MF319 (Microchemicals) and water for 40 s (Fig. 1d).

**Figure﻿ 1:**
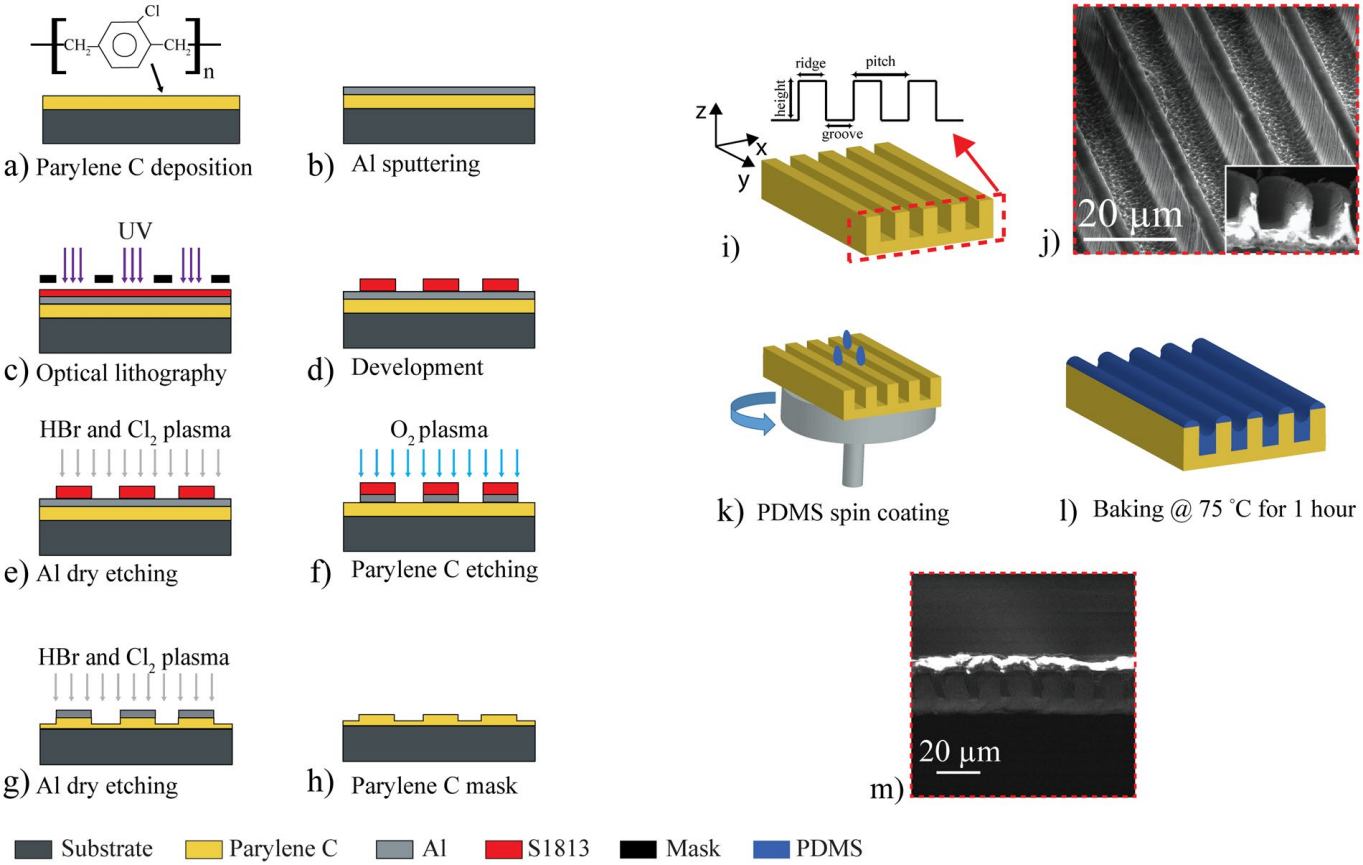
﻿Schematic of the microfabrication process of Parylene C/PDMS hybrid constructs. Microfabrication procedure to obtain high aspect ratio Parylene C grooves (**a**–**i**). A sketch of Parylene C with cross section (dash red line) and the characteristic features (height, ridge, groove and pitch) is illustrated in (**i**). In (**j**) a representative SEM image of only Parylene C is shown. PDMS was spin coated (**k**) and baked at 75 °C for 1 h (**l**). The cross section of the bilayer is shown in the SEM image (**m**).

Al was then dry etched in a plasma of hydrogen bromide (HBr) and chlorine (Cl_2_) in ICP (OPT 100 ICP 380, Oxford Instruments Plasma Technology) for 1 min and 40 s (estimated etching rate ~2 nm/s) at pressure of 5 mTorr, 150 W source power and 800 W bias generator power to create open Parylene areas (Fig. 1e). Parylene etching was conducted in an inductively coupled plasma reactor (ICP, OPT 100 ICP 380, Oxford Instruments Plasma Technology) using O_2_ plasma at pressure of 1.33 Pa, flow 100 sccm, 1000 W source power and 20 W bias generator power (Fig. 1f). The remaining Al was completely removed using the same plasma etching process for 1 min and 45 s (Fig. 1g,h). After this process Parylene C became hydrophobic thus it was mildly treated in oxygen plasma for 15 s before spin coating PDMS. Ridges, grooves, pitches and depth of Parylene C were evaluated by scanning electron microscopy (SEM) of cross section attained from free standing Parylene C membranes cut by scissors (Fig. 1i,j).

PDMS (Sylgard 184) was prepared with a monomer to curing agent ratio of 10:1, mixed for 5 min and degassed for 40 min. It was then spin coated on Parylene C templates using two steps in order to have a conformal layer^21^. PDMS pre-polymer was spun at 500 rpm for 30 s to allow the liquid to cover uniformly Parylene C substrate and then the spin speeds equal to half of the target speed (1000–3000 rpm) was set for 30 s. The final step was determined by the target spin speed (2000–6000 rpm) for 60 s to reach the wrinkled PDMS surface. After spin coating, samples were cured at 75 °C for 1 h and let cool down at room temperature (Fig. 1k). 1cm × 1cm squares samples were cut using a scalpel obtaining a composite free standing membranes (Fig. 1l). Cross section SEM images (Fig. 1m) were taken in order to understand the distribution of PDMS top layer on Parylene C bottom layer.

Flat surfaces were prepared for nanoindentation tests. 13 mm glass coverslips were coated with 8 µm Parylene and 8 µm PDMS as single layers. 8 µm is the thickness of PDMS film spin coated at 6000 rpm. We fixed the thickness of the PDMS since the spin coating can also affect the elastic properties of this material^31^. The sandwich Parylene C/PDMS was prepared by coating 13 mm glass coverslips with 8 µm Parylene C and by spin coating 8 µm PDMS.

### PAm hydrogels preparation and patterning

**Figure 2:**
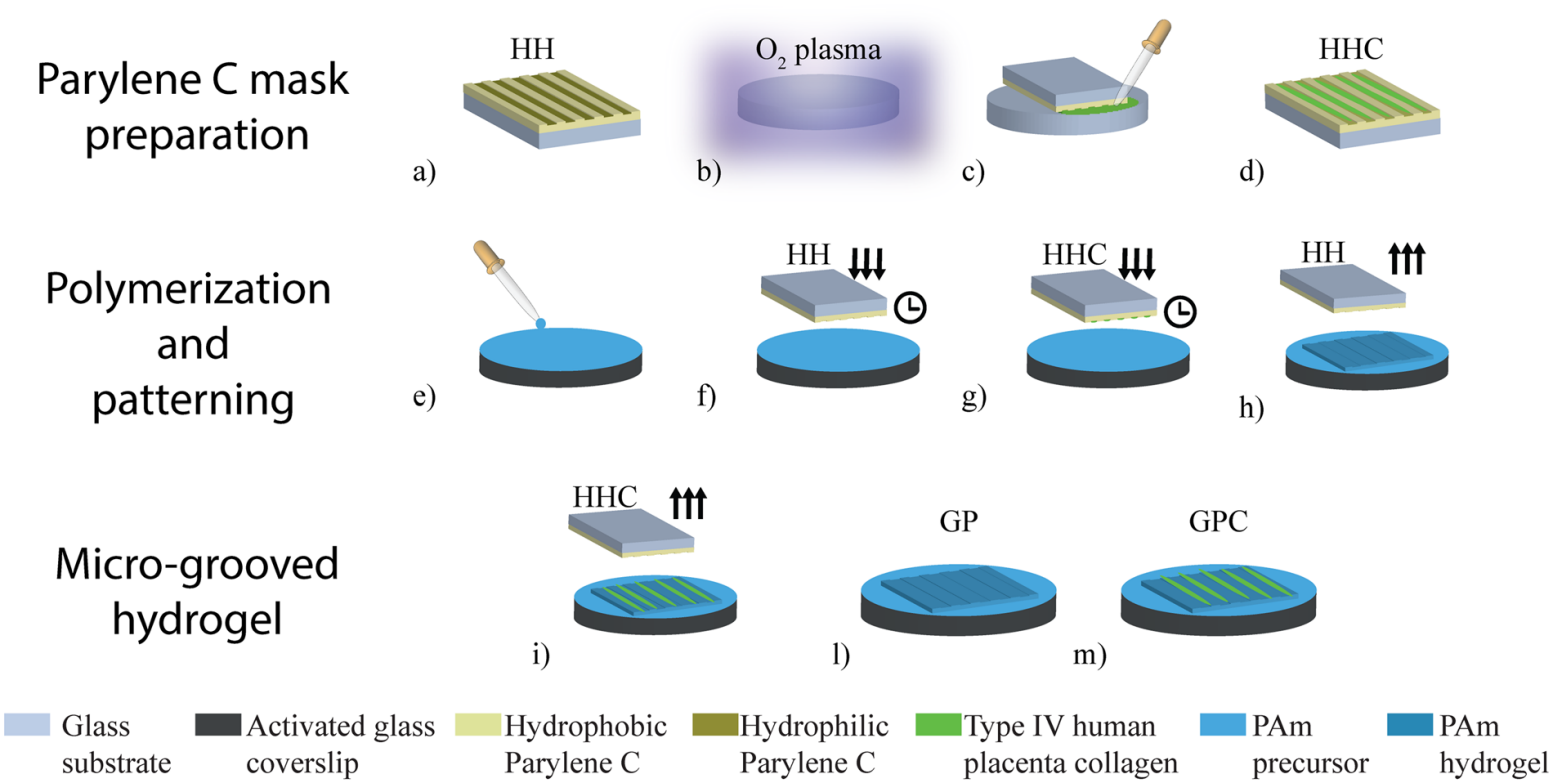
Parylene C preparation and polyacrylamide hydrogel patterning process. HH masks were prepared using standard lithographic process (**a**). Glass was activated using oxygen plasma (**b**) and protein solution was flushed in HH mask microchannels in order to pattern the template and have selectively protein only in Parylene C hydrophylic trenches (**c**) creating HHC masks (**d**). PAm solution was pipetted into silanized coverslips (**e**) and HH and HHC masks were placed upside-down on PAm pre-polymer (**f**,**g**). After gelation time, masks were removed (**h**,**i**) and PAm hydrogel was patterned either topographically (GP) **(l**) and biochemically with collagen (GPC) (**m**).

Hydrophobic/hydrophilic (HH) masks were used as stamp to generate topographically patterned PAm hydrogel. HH masks (Fig. 2a) were fabricated as described elsewhere^6^. Briefly, 5–8 µm thick Parylene C was deposited on 6 inch borosilicate glass wafers succeeded by a spin coating of 1.3 µm thick positive photoresist (S1813, Microchemicals). Wafers were then soft baked at 110 °C for 60 s and exposed to UV light (EVG 620 Mask Aligner) for 2 s through the chrome-plated glass mask consisting of 10 µm-wide and 10-µm spaced features. The samples were developed in MF319 (Microchemicals) and water for 40 s. The etching of Parylene was conducted using O_2_ plasma as described previously. The etching time was 1 min and 40 s for ~1 µm deep grooves.

13 mm glass coverslips were washed in acetone, isopropanol and deionized water. They were firstly treated by oxygen plasma to remove the residual organic particles and to improve wettability of the glass too. Secondly, they were coated with 30 µL of working solution (Bind-silane, General Electric Healthcare Lifesciences) and allowed to react for 3 min in a fume hood. They were then washed with 95% ethanol (EtOH) and dried with nitrogen gun. This chemical modification of the surface provides a better covalent bind of the gel on the glass^32^.

To functionalize the mask with protein, 13-mm glass coverslips were oxidized using oxygen plasma (Fig. 2b). HH masks were put upside down on the functionalized glass slides and 10 µl of protein was spread at the entrance of trenches (Fig. 2c) hollowed in Parylene C. With this procedure HH masks selectively patterned with collagen (HHC) were obtained (Fig. 2d).

Polyacrylamide hydrogel was prepared varying an existing protocol^33^. 2 mL of 40% w/v acrylamide solution (A4058, Sigma-Aldrich,), 2.4 mL of 2% w/v bis-acryalmide solution (Sigma-Aldrich, 146072) and 5.6 mL of water were mixed and degassed for 1 h. 5 µL of 10% w/v ammonium persulfate (APS, Sigma-Aldrich A3678) and 0.5 µL of N,N,N′,N′ Tetramethylethylenediamine accelerator (TEMED, Sigma-Aldrich, T9281) were added to 500 µL of acrylamide/bisacrylamide solution.

PAm hydrogel was obtained by radical polymerization of acrylamide monomer and bis-acrylamide cross-linker with total polymer content equal to 8.48% (w/v) and cross-linker concentration (w/w) equal to 5.66%. 60 µL of polyacrylamide solution was pipetted onto the silanized coverslips (Fig. 2e) and HH and HHC masks were directly printed on polyacrylamide solution still liquid (Fig. 2f,g). After 1 h the gelation of PAm was completed, HH and HHC masks were removed (Fig. 2h,i) having grooved polyacrylamide hydrogel (GP) (Fig. 2l) and grooved polyacrylamide functionalized with collagen (GPC) (Fig. 2m). GP and GPC were washed in water and stored in 1 X phosphate buffer saline (PBS) solution at 4 °C.

### Topographical characterization of the grooves

The average depth of high aspect ratio grooves on Parylene C was measured by a KLA-Tencor Alphastep profiler.

The average values of the ridges, grooves and pitches transferred on PAm gel in hydrated condition were obtained by means of an optical microscope (Zeiss Axio Lab, with objective lenses covering a magnification range from 5X to 50X).

Surface topography of samples in dry condition was analyzed by AFM (Multimode Nanoscope V, Veeco) in no contact tapping mode. Al-coated Tap300 Al-G Si tips (Budget Sensors) with resonance frequency 204–497 Hz and force constant 10–130 N/m were used. 50 µm × 50 µm height images were captured with scan rate of 0.6 Hz and 512 scanning lines. Parylene C/PDMS square samples were cut and peeled-off from the wafer and fixed on AFM sample support discs using carbon tape. Parylene/PDMS constructs, HH masks and GP were analyzed on glass fixed on AFM support discs. AFM images were processed by means of Gwyddion software (http://gwyddion.net/). Surface topography was quantitatively evaluated capturing three height images in three different areas of the same sample and three samples per each category are evaluated. The corresponding average height profiles were traced along the direction perpendicular to the orientation of the grooves and ridges, grooves and depth of the pattern was extracted. The average height of pattern of the mask and of the corresponding gel in dry condition was normalized to the average height of the features (h_y_(x)) either both the stamp and the replica on the hydrogel.

### Nanoindentation tests

Nanoindentation tests were performed by using a Nano-Test Vantage platform 4 (Micro Materials, Wrexham, UK) to evaluate the effect of Parylene C bottom layer on mechanical properties of the composite structure (Parylene C/PDMS). Nanoindentation tests were carried out by using a diamond Berkovich indenter (three sided pyramidal shape with tip radius < 100 nm) in controlled load mode. During the tests, maximum load (P_max_) and loading/unloading rate (0.2 mN/s) were taken constant whereas the penetration of the indenter was continuously recorded in each experiment (15 indentations per each sample). The holding time at maximum load was fixed to 40 s for Parylene C and 60 s for PDMS and Parylene C/PDMS samples in order to have information about time-dependent behaviour (creep) of the indented samples. Creep information were dected during the hold time at constant load. The elasto-plastic response of Parylene C was tested by applying nine constant loads between 0.5 mN to 6 mN. For PDMS on glass 0.1 mN, 0.2 mN, 0.5 mN, 1 mN and 1.3 mN were applied. PDMS/Parylene C composite was tested applying 6 different loads ranging between 1 to 6 mN. Drift measurements were carried out for 60 s during the unloading with a load of 10% of the maximum load. The drift rate was calculated from a linear regression of the displacement vs time data and used to correct nanoindentation data. The Berkovich tip was calibrated before tests by using a standard fused silica sample. Data were corrected for frame compliance before calculation of mechanical prioperties based on the procedure outlined by Oliver and Pharr.

The reduced Young’s modulus (E_r_) was measured at any given load and the Young’s modulus was obtained from he following formula:

$$\frac{1}{{E}_{r}}=\,\frac{1-{\nu }_{s}^{2}}{{E}_{s}}+\frac{1-{\nu }_{i}^{2}}{{E}_{i}}$$

where E_s_ is the Young’s modulus of the sample, E_i_ is the Young’s modulus of the diamond indenter tip (1140 GPa), ν_s_ is the sample Poisson’s ratio and ν_i_ is the Poisson’s ratio of the diamond indenter (0.07). The substrate effect was minimized through the method of least square plotting E_r_ at every single load and extracting the final value ﻿of the Young’s modulus (E) at zero load^34^. The Poisson’s ratio used for PDMS was 0.4^35^ while the one of the bilayer Parylene C/PDMS was 0.5^36^.

### Collagen IV fluorescent labelling

5 mg of type IV human placenta collagen (Sigma-Aldrich, C7521) was reconstituted in 5 mL of phosphate buffer (PBS). The sample was split into 10 0.5 mL aliquots and stored at −20 °C. 1 mg of NHS activated AlexaFluor480 green fluorescent dye (Invitrogen) was dissolved in 200 µL dimethylformamide. Sample was split into 20 10 µL aliquots and stored at −20 °C. 28 µL of AlexaFluor480 (5 mg/mL) was added in a solution of 500 µL of collagen (1 mg/mL). Reactions were allowed to progress on a tube roller for 1 h 15 min at room temperature. Unreacted dye was removed by centrifugal filtration at 3000 RCF, using 10000 MWCO spin filters. Protein was washed three times with water. Dye conjugated collagen was collected from the filter surface after dismantling the filter apparatus by immersion in 1 mL PBS for 5 minutes under agitation (tube roller). Green-labelled protein transferred on HH masks and PAm hydrogels was detected using a fluorescence confocal microscope (Zeiss Axiovert 200).

Fluorescent micrographs of micro patterned FITC-labelled protein were analyzed by using ImageJ software (available at http://rsbweb.nih.gov/ij/download.html) to extract the intensity profiles.

### Statistical Analysis

Analysis of variance of the collected data was performed in order to statistically evaluate the significance differences among samples. A t-test was also performed to compare two groups and the significance was fixed at 5%.

### Data Availability

A﻿ll data supporting this study is available from the University of Southampton repository at: http://doi.org/10.5258/SOTON/D0126.

## Results and Discussion

### Parylene C/PDMS constructs analysis

**Figure 3:**
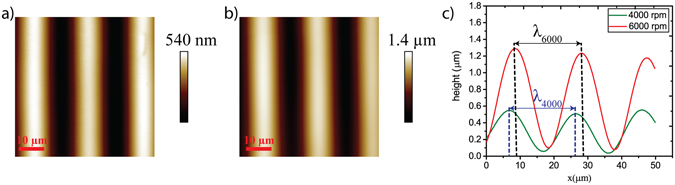
AFM measurements (scan area of 50 µm × 50 µm) at two different target spin speeds and the relative average height profiles. At 4000 rpm (**a**) and 6000 rpm (**b**) AFM images show well defined wrinkled topographies. The average height profiles at 4000 rpm (green) and at 6000 rpm (red) corresponding to the AFM images are shown in (**c**) providing the height and the wavelengths of the grooves at 4000 rpm (λ_4000_) and at 6000 rpm (λ_6000_).

In this work, a sandwich between two polymers is exploited and a new method to pattern PDMS is demonstrated avoiding complex techniques and allowing more elastic substrates. Parylene C was fabricated with depth of the features of 17.65 ± 0.86 µm in order to have wrinkled surfaces made of PDMS. The height of bottom trenches, the spin speeds and the viscosity of the underneath material affect the leveling of upper materials during spin coating^37^. The surface of the hybrid composite Parylene C/PDMS has been analyzed fixing the composition (viscosity), the curing time and temperature of PDMS. Different spin speeds have been exploited and highly anisotropic structures were found only at 4000 rpm (Fig. 3a) and 6000 rpm (Fig. 3b). At 1000 and 2000 rpm the PDMS top layer on micro-grooved Parylene C was found to be flat demonstrating that the PDMS is planarized at low spin speeds.

The average height was extracted from height profiles traced alongside the x direction (perpendicular to the grooves) (Fig. 3c) and it was found to be 459.94 ± 21.32 nm at 4000 rpm and 1.15 ± 0.03 µm at 6000 rpm. The wavelength (λ) of the grooves at 4000 rpm (λ_4000_) is 19.35 ± 0.1 µm and at 6000 rpm (λ_6000_) is 19.55 ± 0.01 µm. The wavelength of the microgrooves is almost 20 µm following the underneath pattern of Parylene C (ridge of approximately 4 µm, groove of almost 14 µm and pitch of almost 19.5 µm). These results are promising for application in TE. The structures could mimic the fibrillar composition of the heart’s ECM and the nano- and microgrooved topography with these characteristics promotes sarcomeric organization and alignement on cardiomyocytes^38^. A composite Parylene C/PDMS we propose has three main advantages. First, this composite construct can be peeled off and become free standing structured elastic membrane. Parylene C is used as a supportive layer that help thin PDMS to be handled and patterned at the same time. Secondly to have PDMS based substrates we do not use materials underneath PDMS that needs to be dissolved in water in order to obtian free standing elastic PDMS membranes^24^. Finally, Parylene C/PDMS is a composite material with elastic anisotropic structures controllable by changing the velocity of the spin coating process.

### Elastic properties

Nanoindentation can be used to study mechanical properties of polymers at micro and nanoscale. Polymers as well as biological tissues present intrinsic heterogeneity in chemical structures that could be probed by nanoindentation^39^.

**Figure 4:**
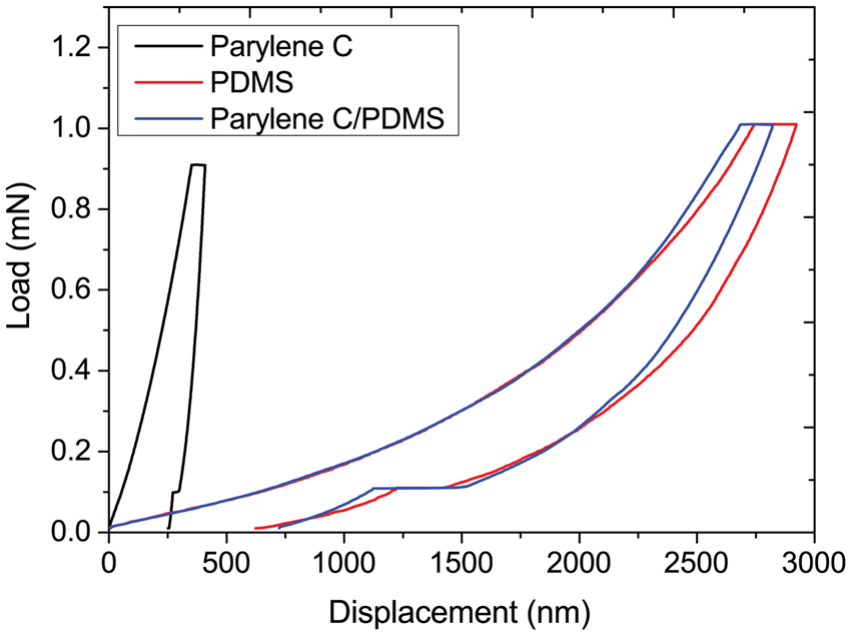
Typical load displacement curves. Comparison of the load-displacement curves of Parylene, PDMS and composite material at maximum load of 1 mN and loading/unloading rate of 0.2 mN/s.

Repeatable load-displacement curves were obtained at different loads with very low scattering. Typical curves of Parylene C at maximum load of 0.9 mN (Fig. 4, black curve), PDMS at 1 mN (Fig. 4, red curve) and composite Parylene C/PDMS (Fig. 4, blue curve) are reported. The comparison of the curves obtained for the three materials show that the Parylene C has a less elastic behaviour than PDMS and Parylene C/PDMS. In addition, the PDMS constructs and the bilayer Parylene C/PDMS show a very similar behaviour. The maximum displacement (h_max_) at maximum load, the permanent depth (h_f_) when the indenter is unloaded and the Young’s modulus were extracted. As shown in Table 1 under the same load Parylene C has a lower value of h_f_ and h_max_ than the other two samples.

**Table 1:** Mechanical properties of the constructs.

Material	h_f_ [nm]	h_max_ [nm]	h_f_/h_max_	E_s_	E
Parylene C	256.04 ± 18.4^a^	446.31 ± 89.4^a^	0.57^a^	4.35 ± 0.4^a^ [GPa]	4.85[GPa]
PDMS	895.90 ± 93.4^b^	2815.84 ± 72.2^b^	0.33^b^	55.46 ± 1.9^b^ [MPa]	22 [MPa]
Parylene C/PDMS	723.33 ± 184.4^b^	2818.25 ± 60.8^b^	0.26^b^	59.33 ± 2.4^b^ [MPa]	47.3 [MPa]

^a^Values for 0.9 mN. ^b^Values for 1 mN.

Parylene C have also presented an anhomalous behaviour when the Young’s modulus vs load was plotted. In particular, a polinomial fit was found for all applied loads. The Young’s modulus of Parylene C drops sharply at loads between 0.5 mN to 3 mN and it starts to increase from 4 mN to 6 mN. The linear fit was obtained using loads ranging from 0.5 mN to 1.8 mN and the effective Young’s modulus at zero load was evaluated to be 4.85 GPa. The value of the Young’s modulus of the sample extracted using the formula with the reduced Young’s modulus is also reported in Table 1. It was estimated to be 4.35 ± 0.38 [GPa]. Thin PDMS was also tested and it resuled softer than the Parylene C since the curve shows a high maximum displacement of almost 2.9 µm at 1 mN. The Young’s modulus of the sample was found to be 55.46 ± 1.9 [M﻿Pa] and the value without the effect of the substrate is 22 [MP﻿﻿a] as shown in Table 1.

Nanoindentation tests on the composite material displayed a maximum dispalcement close to the single PDMS and a Young’s modulus two times higher than the single PDMS. These results demonstrate that Parylene C affects the elasticity of PDMS resulting in a composite construct more elastic than the single Parylene C.

The material around the contact area deforms elastically during the indentation (sink-in effect^40^) with the ratio h_f_/h_max_ below 0.7. Parylene C showed values below 0.7 for loads ranging from 0.5 mN to 1.8 mN and higher than 0.7 (pile-up effect^40^) at 2.5 mN and 6 mN respectively demonstrating a plastic deformation. At 1.3 mN and 1.8 mN the ratio h_f_/h_max_ resulted constant and the higher value closed to 0.8 was found at maximum load of 6 mN. In Table 1 is shown the value of h_f_/h_max_ at 0.9 mN. As expected, PDMS have shown a more elastic effect with h_f_/h_max_ almost constant and equal to 0.3 (as indicated in Table 1).

The elastic behaviour of Parylene C/PDMS is evident not only into the values of the Young’s modulus (59.33 ± 2.4 [MPa] and 47.3 [MPa] with and without the effect of the substrate) but also into the ratio h_f_/h_max_ that was found to be lower than 0.7 (Table 1) and it additionally became higher with increasing of the load. When the load increases the plasticity of the beneath Parylene C starts to influence the elastic behaviour of the PDMS top layer as demonstrated from h_f_/h_max_ ratio that ranges from 0.26 at 1 mN (Table 1) to 0.43 at 6 mN. The elastic recovery of the materials can be evaluated calculating the fraction of deformation coefficient (η) as (h_max_ − h_f_)/h_max_. This value was found to be 0.43, 0.66, 0.74 for Parylene C, PDMS and Parylene C/PDMS respectively. The lowest value of Parylene C indictaes that the Parylene C is not totally plastic but elasto-plastic and recover the original depth for almost 50%.

The elastic work (W_E_) was given as elastic recovery of the material during the unloading whereas the plastic work (W_p_) was evaluated as the difference between the area under loading and unloading curves. W_E_ and W_P_ for Parylene C at 0.9 mN resulted 0.05 ± 0.005 nJ and 0.13 ± 0.05 nJ respectively. The W_E_ of PDMS and Parylene C/PDMS samples at 1 mN resulted almost the same equal to 0.6 nJ, confirming the elastic behaviour of PDMS and of the composite Parylene C/PDMS.

In addition, creep analysis showed that the time-dependency is not so evident for the three constructs and the creep is not affecting the results. In addition, PDMS and Parylene C/PDMS show a slight difference in creep under the same load demonstrating that they have a similar behaviour.

### Polyacryalmide patterning

**Figure 5:**
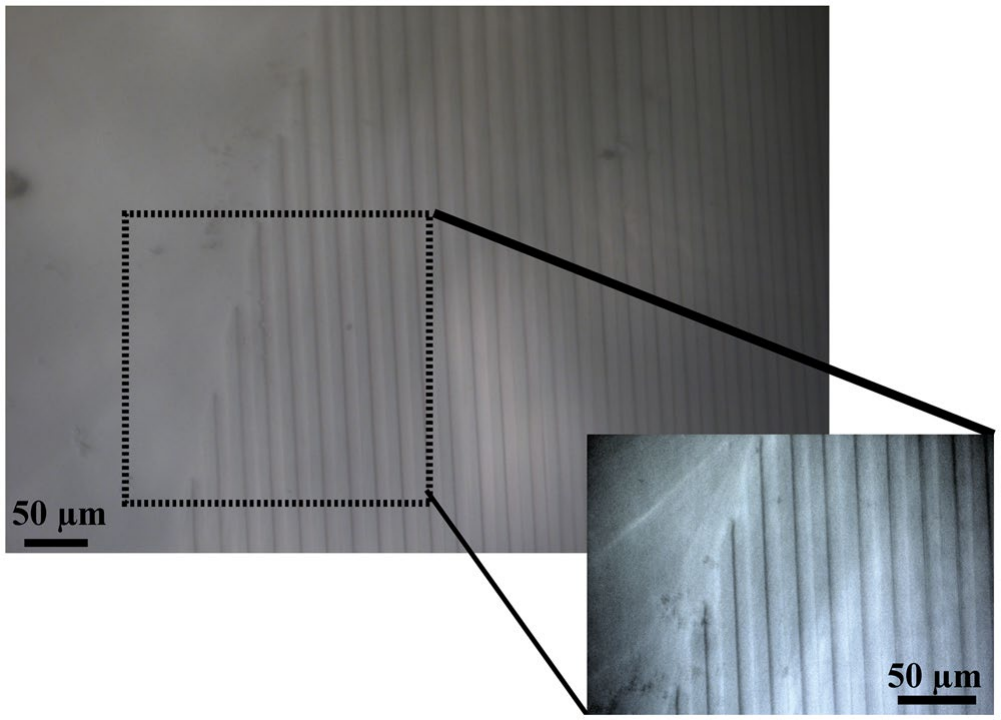
Optical micrographs of micro-patterned hydrated PAm hydrogel.

The topographic pattern on PAm was obtained by the combination of the pressure of the mask and the capillarity of the liquid into the mask. The pattern of the gel in wet conditions was evaluated using optical microscopy (Fig. 5). The average values of the ridges and the grooves were found to be 7.85 ± 1.24 µm and 10.12 ± 1 µm in comparison to the values of the ridges and grooves of the mask that were 6.32 ± 0.1 µm and 6.88 ± 0.6 µm respectively.

**Figure 6:**
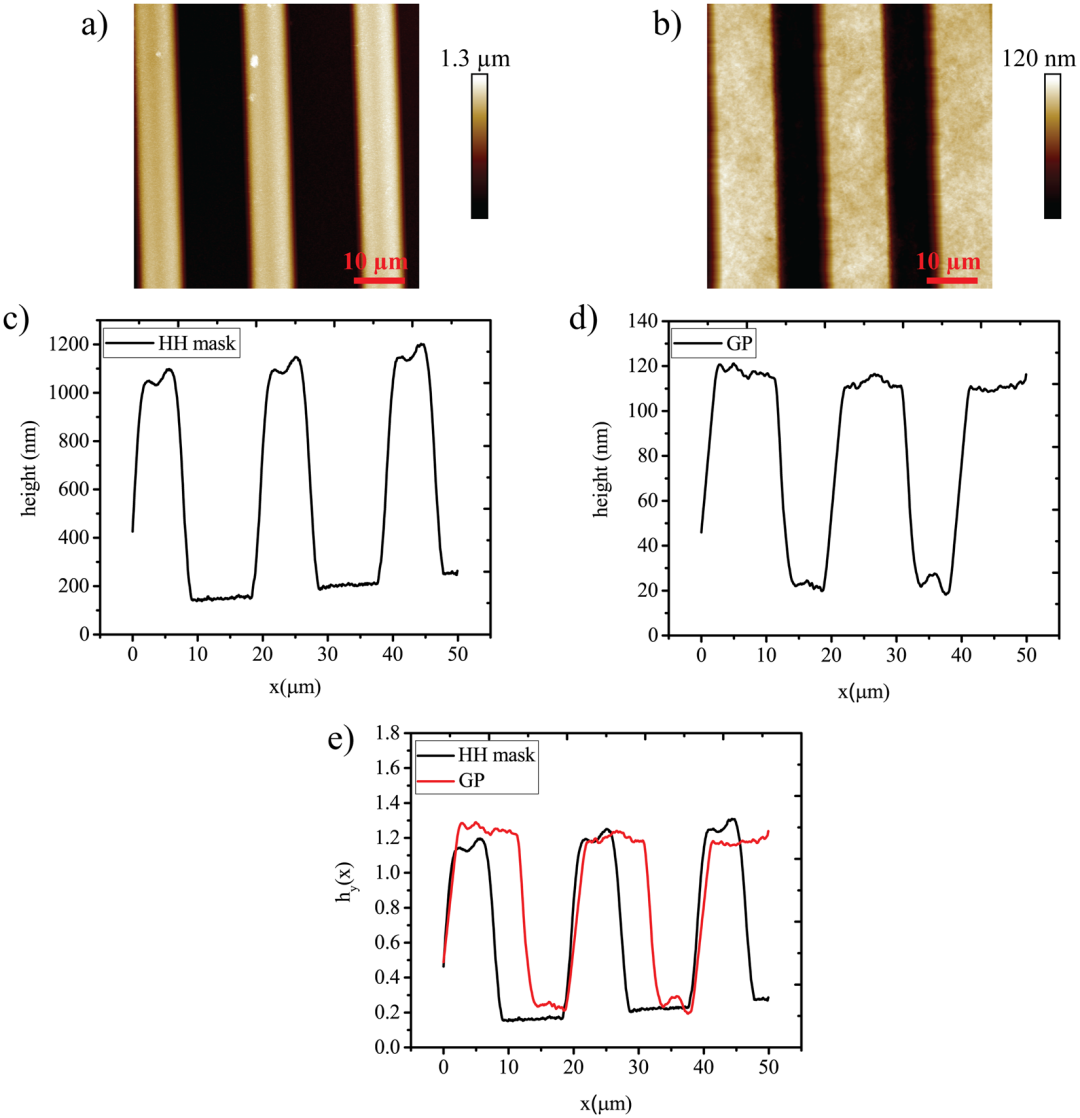
AFM image of Parylene C HH mask and GP replica with relative profiles of the grooves. AFM measurements of the mask and of the hydrogel are compared (**a**,**b**) with the correspondig height profiles averaged on the same image (**c**,**d**). The normalized height profiles of HH mask and of GP are compared (**e**) in order to demonstrate that the micro-grooves of the gel follow the same trend of the mask.

AFM meausrements in dry condition were taken on H﻿H ma﻿sks and on PAm hydrogel to demonstrate the retention of the features of the HH masks (Fig. 6a) on GP substrates (Fig. 6b). The corresponding average height profiles of HH molds and of GP hydrogels were plotted alongside the features and they are shown in Fig. 6c,d. Three repetition were carried out and no significant difference was found out between the samples. The average height of the features of the HH mask measured by AFM was found to be 974.18 ± 0.1 nm and the average height of the features of the GP was found to be 88.12 ± 15.1 nm. The height of the features cannot be compared since in dry condition the gel de-swell loosing water. The average values of the ridges of the mask and of the grooves of the gel were compared and there was no significant diffence between them with p > 0.05 (5.85 ± 1.4 µm for the mask and 5.45 ± 3 µm for the gel). Either the grooves of the mask and the ridges of the gel were compared with the corresponding values of 8.6 ± 1.6 µm and 5.4 ± 2.9 µm rispectively. The height profiles were normalized for the average height for both the gel and the mask as shown in Fig. 6e in order to evaluate the trend of the pattern of the mask and of the gel. The maximum value of h_y_(x) is reltively equal whe﻿the﻿r for the mask or for the gel with a value of almost 1.3. With these results we can confirm that the patterning process is repeatable and the features of the mask are faithfully transferred on the hydrogel.

**Figure 7:**
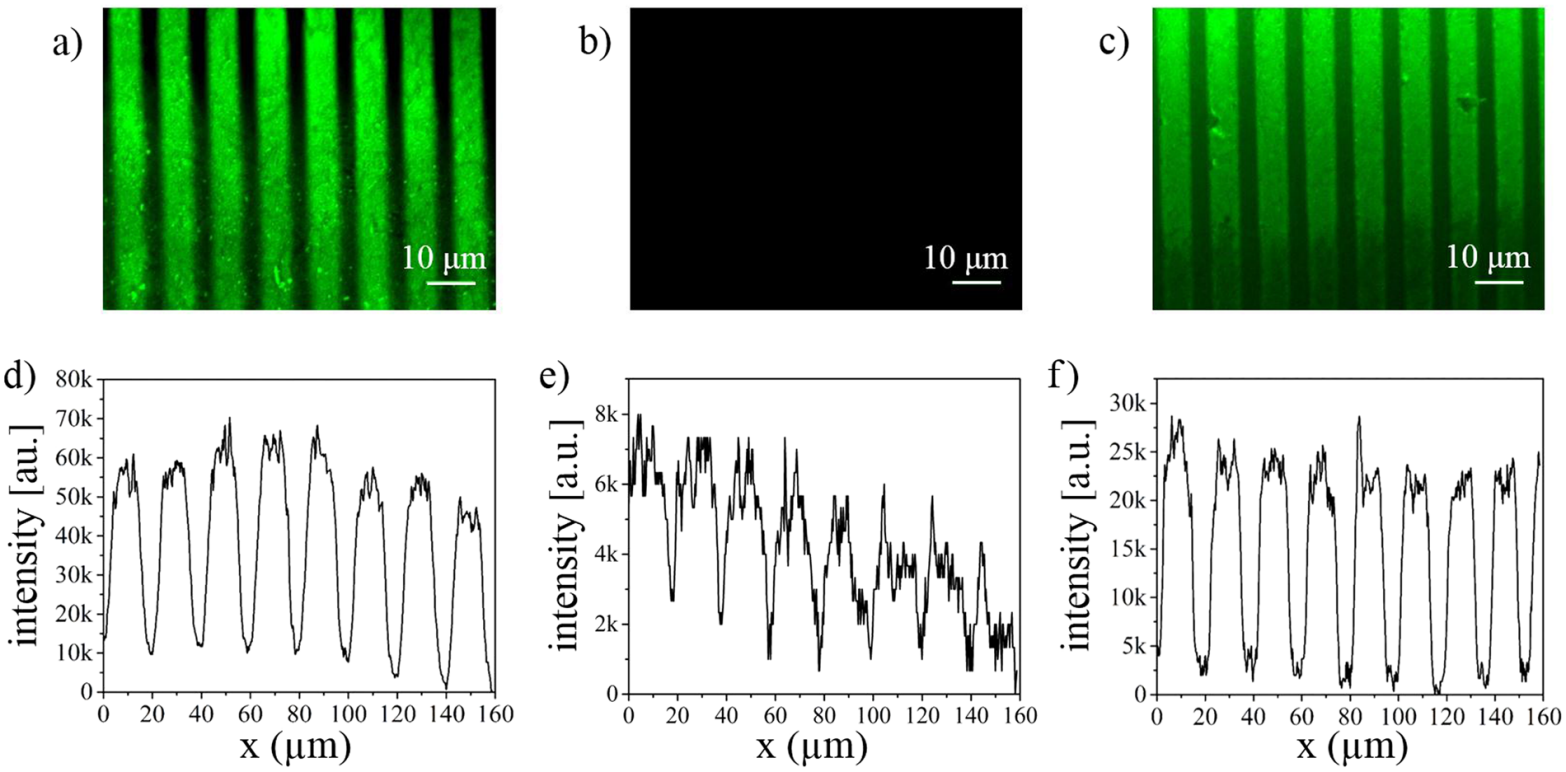
Fluorescence images of protein patterned PAm hydrogel and averaged intensity profiles. Collagen patterned on HH masks (**a**), no protein on the mask after the patterning (**b**) and protein totally transferred on PAm gel. All the images were captured at the same exposure time (200 ms).

Since the hydrophobic/hydrophilic selectivity of Parylene C was studied previously and it was revealed that protein can be absorbed only in hydrophylic channels for capillary effect^6^, we have used this property to pattern collagen IV and subsequently pattern PAm hydrogel. Thanks to the properties of Parylene C masks we have demonstrated that it is possible to transfer proteins directly on PAm hydrogel without any complicated strategy reducing the steps normally used in PDMS-based micro contact printing^41^. During the gelation the protein could be entrapped into the gel since the mask is printed directly on the gel still liquid which could have hydrophilic interaction with the protein. Thanks to the stiffness and the impermeability property of the Parylene the gel does not stick on the mask. The mask was evaluated before (Fig. 7a) and after the printing (Fig. 7b) by fluorescence microscopy. The mask showed bright fluorescence before printing (Fig. 7a), and it resulted almost completely dark after the printing (Fig. 7b). The protein was transferred onto the gel. Indeed, collagen fluorescent lines are clearly visible in Fig. 7c where it is evident that the protein was patterned on the hydrogel.

The averaged intesity profiles plotted along the direction normal to the grooves clearly show how the fluorescence intensity reached its highest (~70 k a.u.) on the grooves in the mask before printing (Fig. 7d), whereas the maximum intensity was around 28 k a.u. in the patterned gel with the transferred protein (Fig. 7f). Reasonably the fluorescence was very low (up to 8 k a.u.) on the mask after printing (Fig. 7e) meaning that there was not much protein left on the mask.

## Conclusions

In conclusion, we have presented two new techniques using Parylene C as a mask to pattern soft materials particularly PDMS and PAm hydrogel. PDMS was patterned topographically using high aspect ratio Parylene C grooved substrates. PDMS showed a wavy topography that was controllable by changing the velocity of the spin coating. With this method it was possible to have a new elastic composite material that presented all the possible requirements for application as scaffold in TE. Results showed that the wavelength of the grooves was following the pattern of the underneath Parylene C with different height at two different spin speeds.

Parylene C was also used as template for patterning topographically and biochemically PAm hydrogel demonstrating that it is possible to pattern the hydrogel using selectively modified Parylene C. The topographic pattern was successfully transferred on the hydrogel with well-defined features. Protein transfer on PAm hydrogel was also demonstrated with fluorescence analysis and the protein was found to be on the hydrogel following the pattern of the hydrophilic strips of the Parylene C mask.

Therefore the well-defined topographic pattern and protein transfer on PAm hydrogel may be considered as a method to create new scaffolds to study the effect of both topography and elasticity of *in-vitro* cell cultures.
